# Analysis of the Screening Results for Congenital Adrenal Hyperplasia Involving 7.85 Million Newborns in China: A Systematic Review and Meta-Analysis

**DOI:** 10.3389/fendo.2021.624507

**Published:** 2021-04-23

**Authors:** Zhuoguang Li, Lianjing Huang, Caiqi Du, Cai Zhang, Mini Zhang, Yan Liang, Xiaoping Luo

**Affiliations:** ^1^ Department of Pediatrics, Tongji Hospital, Tongji Medical College, Huazhong University of Science and Technology, Wuhan, China; ^2^ Department of Endocrinology, Shenzhen Children’s Hospital, Shenzhen, China

**Keywords:** neonatal screening, incidence, congenital adrenal hyperplasia, 17-OHP, meta-analysis

## Abstract

**Background:**

Congenital adrenal hyperplasia (CAH) is a group of congenital genetic diseases caused by defective steroidogenesis. Our study aims to systematically analyze the screening results for CAH in Chinese newborns.

**Methods:**

Studies were searched from PubMed, Web of Science, Cochrane library and some Chinese databases up to September, 2020. Meta-analysis was performed after quality assessment and data extraction.

**Results:**

After a review of 2 694 articles, we included 41 studies enrolling 7 853 756 newborns. In our study, we found that the incidence of CAH in China was 0.43‱ [95% confidence intervals(CI), (0.39‱, 0.48‱)], or 1/23 024 [95%CI, (1/25 757,1/20 815)]. 27 studies were included for analysis of the screening positive rate, which gave a rate of 0.66% [95%CI, (0.54%, 0.78%)]. As for the recall rate of positive cases, 17 studies were included and showed that the recall rate reached 86.17% [95%CI, (82.70%, 89.64%)]. Among the CAH patients, the ratio of males to females was 1.92:1 (119:62), and the ratio of salt wasting (SW) to simple virilization (SV) type was 3.25:1 (104:32). The average 17-hydroxyprogesterone (17-OHP) value of CAH was 393.40 ± 291.85 nmol/L (Range 33-1 300 nmol/L); there was no significant difference between male and female patients (437.17 ± 297.27 nmol/L v.s. 322.25 ± 293.04 nmol/L, *P*=0.16), but a significant difference was found between SW and SV patients (483.29 ± 330.07 nmol/L v.s. 73.80 ± 7.83nmol/L, *P*=0.04).

**Conclusion:**

We systematically analyzed the current situation of neonatal CAH screening in China, which will deepen our understanding for future CAH screening and early diagnosis.

## Introduction

Congenital adrenal hyperplasia (CAH) is a group of autosomal recessive inherited diseases caused by defects of essential enzymes in the synthesis of steroid hormones. Because of different degrees of aldosterone and cortisol deficiency, classical CAH mainly manifests with salt-wasting symptoms and SV type mainly with hyperandrogenism. Many studies have shown that CAH patients often have some adverse outcomes during childhood or adulthood (1, 2). Therefore, early screening, early diagnosis and early treatment are particularly critical to help patients with CAH to have normal and healthy development.

About 90-95% of CAH cases are caused by deficiency of steroid 21-hydroxylase (21-OHD), characterized by elevated 17-hydroxyprogesterone (17-OHP) and reduced glucocorticoid levels. The current screening for CAH is still dominated by 21-OHD, although some rare types such as 11β-hydroxylase and 3β-hydroxysteroid dehydrogenase deficiency may also be found. Screening for CAH was first performed in United States of America in 1977, and currently more than 35 countries have carried out CAH screening (1, 2). In China, such a screening program started in the early 1990s, and to date, many screening centers have obtained regional incidence data. However, due to China’s vast territory and unbalanced medical provision, CAH screening coverage rate in China was only 18.9%-19.9% according to the statistics of newborn screening in 2013 (3). In addition, there were significant differences in reports of the incidence of CAH, for example, the 2016 CAH guideline (China) stated that the domestic incidence was 1/16 466-1/12 200 (1), while the 2018 Endocrine Society CAH guideline stated that the incidence of CAH in China was as high as 1/6 064 (sample size 30 000 cases) (2). National newborn screening is the only way of obtaining precise incidence data of CAH in China and promote its early diagnosis, but currently there are still many difficulties in carrying out such a national screening program.

Therefore, we used the method of meta-analysis to comprehensively analyze the results of CAH newborn screening in different regions of China, and conducted a systematic analysis of its screening positive rate, recall rate and incidence of CAH. Our study will help us understand the screening status and promote an effective CAH neonatal screening program in the future.

## Methods

### Data Sources and Searches

We developed a protocol for the meta-analysis and followed the principles of the PRISMA statement (see **Supplementary Table 1**). Relevant studies were searched from PubMed, Web of Science, Cochrane library and some Chinese databases (CNKI, Wanfang, VIP and CBMD) up to September, 2020. Our searches were based on combinations of the following index terms: newborn screening, congenital adrenal hyperplasia, CAH, 17-hydroxyprogesterone, 17-OHP or 17α-OHP and the corresponding terms in Chinese. We also reviewed the reference lists of retrieved studies and review articles.

### Eligibility Criteria and Exclusion Criteria

The studies would be included if they met following criteria: (1) Results of CAH newborn screening in different provinces, cities and autonomous regions of China; (2) Sample collection was subject to “Technical Specifications for Blood Collection for Neonatal Disease Screening(China)” or the regional handbook (72 hours after the birth, blood is collected from the inside or outside of the heel to form dried blood spots, which are naturally dried and stored in a refrigerator at 2-8°C, and then sent for testing); (3) Detection methods: dissociation-enhanced lanthanide fluorescence immunoassay (DELFIA) or enzyme-linked immunosorbent assay (ELISA) was used to quantitatively measure 17-OHP values of dried blood spots (Most Chinese laboratories recognize 30 nmol/L as the positive cut-off value, only the Children’s Hospital of Shanghai Jiaotong University takes 40 nmol/L as the cut-off value); (4) The main indicators are the incidence of CAH, the positive rate, the recall rate and some other characteristics related to CAH.

The following exclusion criteria were applied: (1) Studies with overlapping screening regions or screening time; (2) Not meeting the requirements of the eligibility criteria; (3) Studies with low quality. In addition, studies which were not published in English or Chinese were also excluded because of language limitations.

### Data Collection and Quality Assessment

According to the above eligibility criteria and exclusion criteria, a data extraction table was developed and relevant data were collected. The information included: authors, published year, screening year and participants, positive cases and positive rate, recall cases and recall rate, diagnosed cases and their characteristics (gender, clinical classification and 17-OHP levels), etc.

An 11-item checklist recommended by the Agency for Healthcare Research and Quality of America (AHRQ) (see **Supplementary Table 2**) was used to evaluate the quality of included studies. An item would score “0” with answer “NO” or “UNCLEAR”; otherwise, it would score “1”. With a total score of 11 points, article quality was assessed as follows: low quality = 0-3, moderate quality = 4-7, high quality = 8-11. Two reviewers individually assessed the quality of eligible studies, and a senior investigator resolved the discrepancies if necessary.

### Summary Measures and Synthesis of Results

We used the Stata 12.0 software to analyze the data. If different units were used in the studies, they were converted to international standard units. The effect size in our study was shown as “rate” and its 95% confidence interval (95% CI). I^2^ and Chi^2^ tests were used to estimate the heterogeneity, with I^2^ value less than 50%, heterogeneity was considered to be small and a fixed effect model was used; otherwise, the random effect model was used. Subgroup analysis was also conducted to identify the possible sources of heterogeneity. Publication bias was shown by a funnel plot and evaluated by the Begg’s test. Independent sample t test was used for statistical analysis, *P*<0.05 indicated that the difference was statistically significant.

## Results

### Study Selection

**Figure 1:**
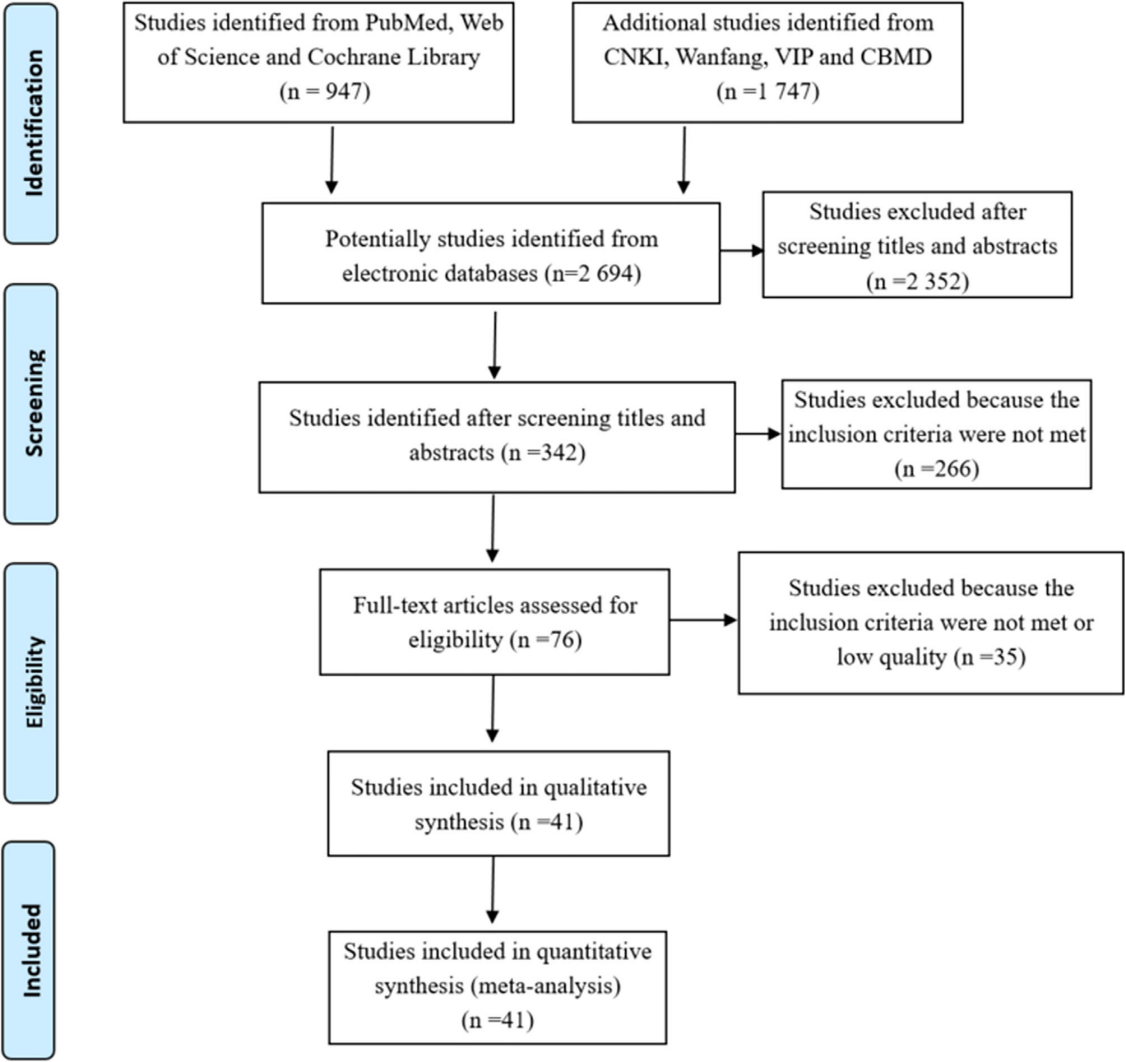
Flowchart depicting literature search and selection (Follow the PRISMA Flow Diagram, for more information, visit www.prisma-statement.org).

Our initial data search yielded a total of 2 694 articles (1 747 articles in Chinese and 947 in English). 2 352 articles were excluded by reading the titles and abstracts, and 266 were excluded because they didn’t meet the eligibility criteria, whereas the remaining 76 were considered as potentially eligible for our analysis. After careful reading of the entire full text, 41 articles with moderate or high quality met the eligibility criteria and were included in the meta-analysis. A flow diagram (**Figure 1**) shows the flow chart of the literature search.

### Quality Assessments

In our included studies, the collection of specimens abided by the “Technical Specifications for Blood Collection for Neonatal Disease Screening” or the guidelines of the corresponding region; the DELFIA or ELISA method was used to detect the 17-OHP concentration of dried blood spot specimens; the main indictors were incidence rate, the positive rate and recall rate of screening, etc.

Based on AHRQ quality assessment items, 41 studies (4–44) that scored four or more were deemed as moderate or high quality. The average score of 7.7 indicated minimal risk of bias. The results are shown in **Table 1** and **Supplementary Table 2**.

**Table 1:** Characteristics of studies included in the meta-analysis.

Area		Years	Screening Cases	CAH cases	Incidence	Positive cases (rate %)	Recalled cases (rate %)	Male : Female	SW : SV	AHRQ scores
Province	City									
Taiwan (4)		2000-2001	192 687	13	1/14 822			6: 7	9: 4	8
Shanghai (5)		2007-2008	93 971	5	1/18 794	214(0.23)	176(82.24)	4: 1		9
Hunan (6)		2009-2013	40 988	4	1/10 247	1 192(2.91)	1 120(93.96)	2: 2	1: 3	9
Guangxi (7)		2012-2015	378 252	22	1/17 193	1682(0.44)				7
Ningxia (8)		2014-2016	160 046	11	1/14 550	70(0.04)	70(100)	6: 5	9: 2	9
Beijing (9)		2014-2017	22 632	2	1/11 316	156(0.69)		2: 0		7
Sichuan (10)		2015-2018	271 283	16	1/16 955				14: 2	9
Shanxi (11)		2015-2016	64 378	3	1/21 459	323(0.50)	297(91.95)	2: 1		9
Zhejiang	Ningbo (12)	2014	88 406	3	1/29 469	517(0.58)		2: 1	3: 0	8
	Others (13)	2014-2016	1 719 510	69	1/24 920					6
Shandong	Jinan (14)	2003-2011	88 350	11	1/8 032			10: 1	9: 2	8
	Taian (15)	2010-2012	161 337	8	1/20 167	1 401(0.87)	1 386(98.93)			8
	Liaocheng (16)	2009-2010	76 383	5	1/15 277	1 456(1.91)	1 235(84.82)			5
	Linyi (17)	2009-2013	740 730	24	1/30 864			12: 12		7
	Heze (18)	2013	119 560	3	1/39 853					5
	Zibo (19)	2010-2014	178 577	11	1/16 234	2 875(1.61)	2 687(93.46)	7: 4	7: 4	9
	Weifang (20)	2012-2015	305 879	14	1/21 849	3 448(1.13)	3 354(97.27)	11: 3	11: 3	8
	Rizhao (21)	2012-2014	101 161	9	1/11 240					5
	Qingdao (22)	2013-2017	566 395	32	1/17 700	2 536(0.45)	2 310(91.09)	22: 10		9
Guangdong	Zhongshan (23)	2008-2010	105 320	2	1/52 660	307(0.29)	168(54.72)	2: 0		9
	Foshan (24)	2010-2011	74 791	5	1/14 958	260(0.35)			2: 3	9
	Shenzhen (25)	2010-2011	329 135	15	1/21 942	1 581(0.48)	1 113(70.40)		13: 2	9
	Dongguan (26)	2009-2013	551 538	17	1/32 443	2 757(0.50)	2 453(88.97)	11: 6	11: 6	9
	Heyuan (27)	2014-2016	45 000	4	1/11 250					7
Jiangsu	Nanjing (28)	1993-2002	103 935	5	1/20 787	401(0.39)		3: 2		8
	Wuxi (29)	1992-2006	61 284	4	1/15 321			3: 1		8
	Changzhou (30)	2001-2010	175 876	13	1/13 529					8
	Suzhou (31)	2010-2012	96 423	5	1/19 285	864(0.90)	464(53.70)	4: 1	4: 1	8
	Yancheng (32)	2012-2014	199 612	9	1/22 179	366(0.18)				9
	Lianyungang (33)	2016	53 305	3	1/17 768	265(0.50)	265 (100)	2: 1	3: 0	9
	Yangzhou (34)	2013-2017	88 829	4	1/22 207	240(0.27)	238(99.17)	3: 1	4: 0	9
Jiangxi	Nanchang (35)	2011-2013	27 988	2	1/13 994	448(1.60)	379(84.60)	2: 0	2: 0	9
	Jiujiang (36)	2015-2017	25 000	3	1/8 333	29(0.12)				7
	Yichun (37)	2016-2017	80 305	4	1/20 076	132(0.16)	112(84.85)	3: 1		9
Chongqing	Yuzhong (38)	2012-2017	125 320	7	1/17 903					5
	Others (39)	2012-2017	25 958	1	1/25 958	21(0.08)				5
Liaoning	Shenyang (40)	2013-2014	23 279	2	1/11 640			0: 2	2: 0	8
Hubei	Shiyan (41)	2016-2017	70 937	3	1/23 646	308(0.43)	299(97.08)			6
Shaanxi	Baoji (42)	2011-2015	192 469	5	1/38 494					9
Fujian	Fuzhou (43)	2013	15 136	1	1/15 136	76(0.50)	70(92.11)			5
Yunan	Kunming (44)	-2007	11 791	2	1/5 896					6

### Study Characteristics

After quality assessments, 41 studies (4–44) with 7 853 756 newborns were included, and 381 cases were diagnosed with CAH (see **Table 1** and **Figure 3**). Of the screened newborns, 95% (except part of Ningxia and Sichuan province) were located to the east of the Heihe-Tengchong line (an imaginary line that divides the area of China into two roughly equal parts with contrasting population densities; west of the line: 57% of the area, but only 6% of the population; east of the line: 43% of the area, but 94% of the population). Among them, the sex ratio of the screened newborns described in our studies was 1.10:1 (1 678 399: 1 527 300). We found that the ratio of males to females with CAH described in some studies was 1.92:1 (119:62), while the ratio of SW to SV type was 3.25:1 (104:32). The average level of 17-OHP (n=74) for patients diagnosed with CAH was 393.40 ± 291.85 nmol/L (Range 33-1 300 nmol/L), there was no significant difference between patients of different genders [male(n=36): 437.17 ± 297.27 nmol/L (Range 33-1 300 nmol/L) v.s. female(n=22): 322.25 ± 293.04 nmol/L (Range 33.2-1 040 nmol/L), *P*=0.16], but a statistical difference was found between SW and SV type [SW(n=25): 483.29 ± 330.07 nmol/L(Range 48-1 300 nmol/L) v.s. SV(n=3): 73.80 ± 7.83 nmol/L (Range 65-80 nmol/L), *P*=0.04].

### Results of Meta-Analysis

#### Incidence of CAH

**Figure 2:**
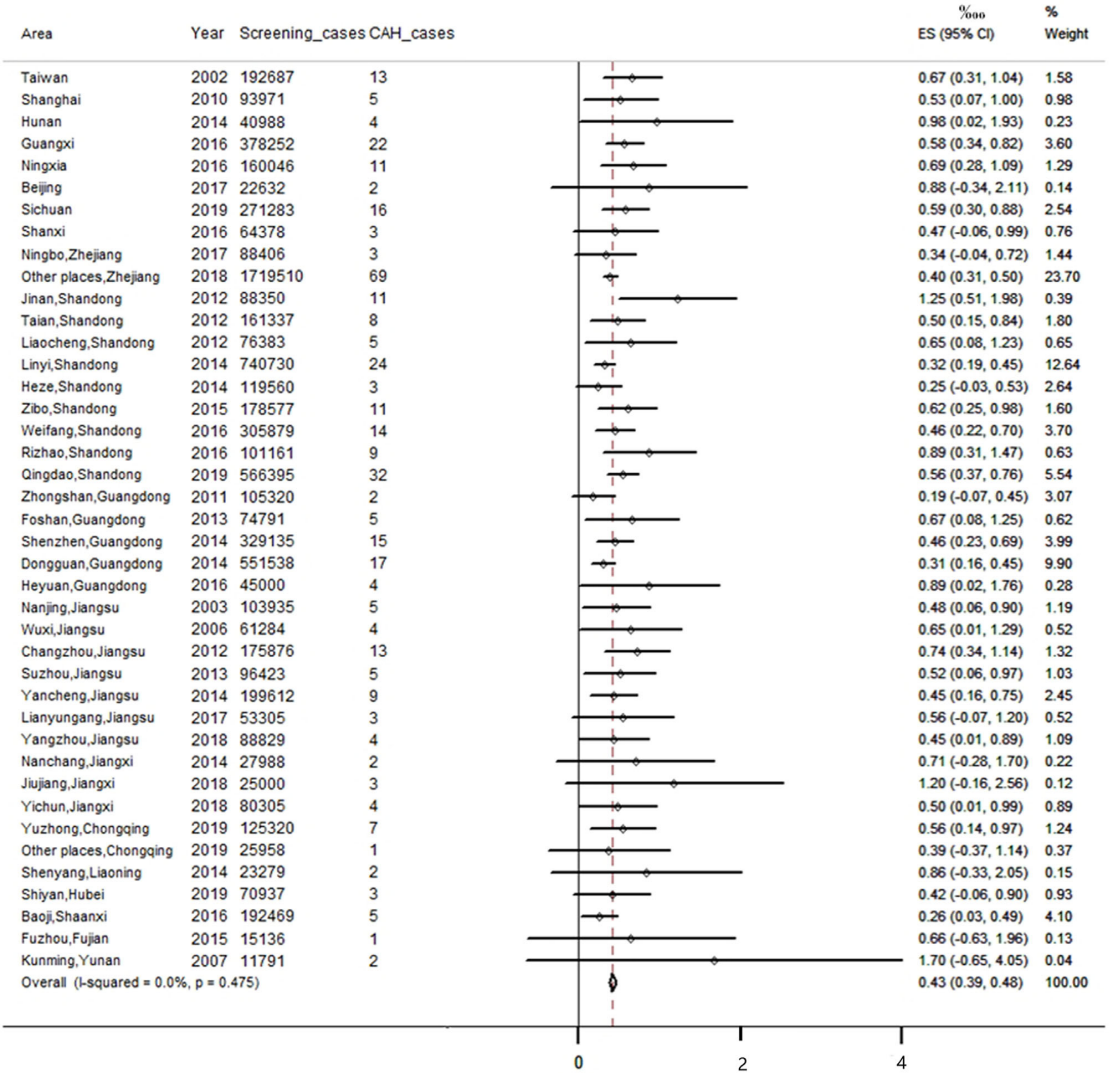
Meta-analysis of CAH incidence in different regions of China.

**Figure 3:**
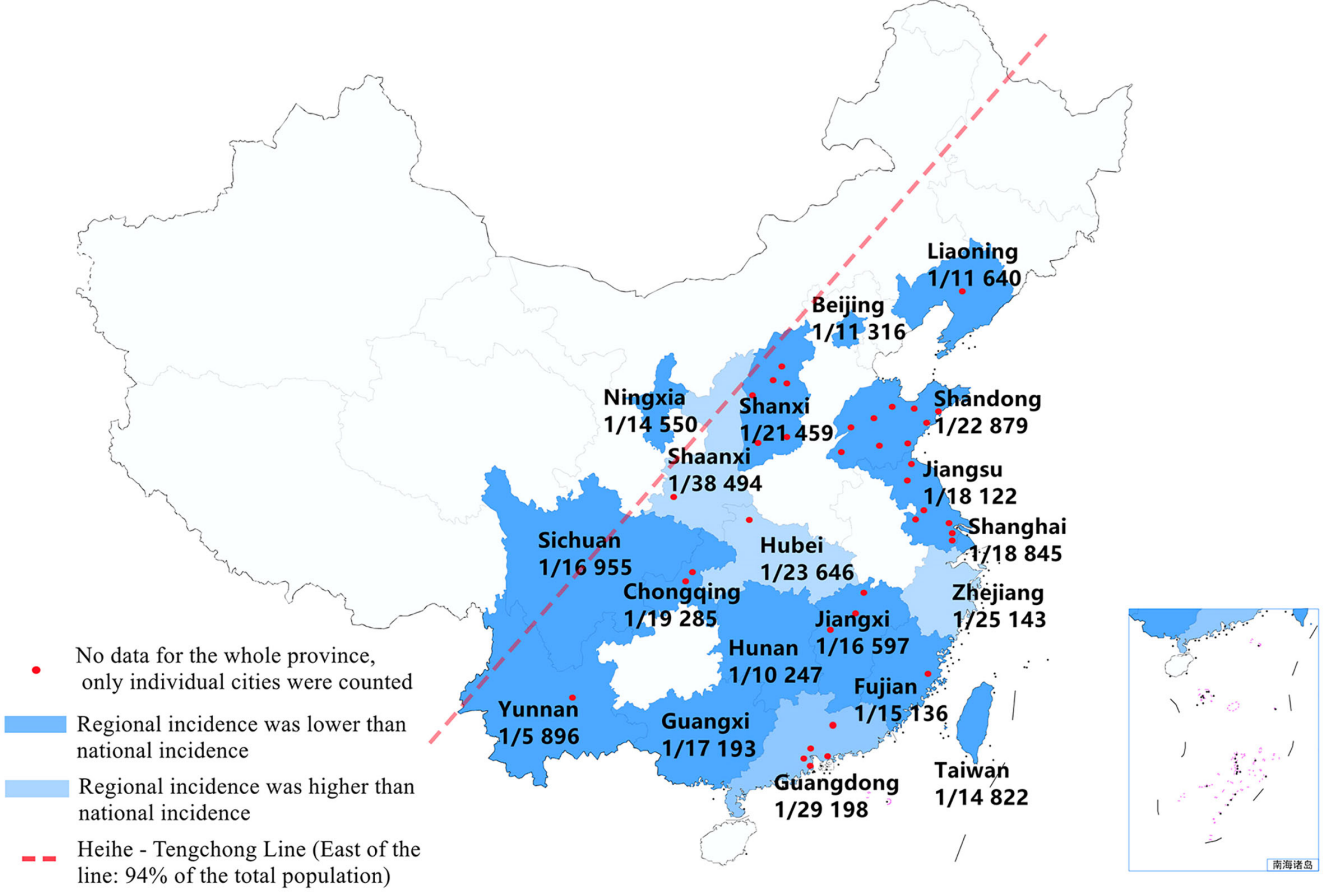
The schematic diagram shows the incidence of CAH in different provinces of China (provincial data obtained by subgroup analysis). Note: Of the screened newborns, 95% cases (except part of Ningxia and Sichuan province) were located to the east of the Heihe-Tengchong line (an imaginary line that divides the area of China into two roughly equal parts with contrasting population densities; west of the line: 57% of the area, but only 6% of the population; east of the line: 43% of the area, but 94% of the population).

In the included studies, 41 studies reported the incidence of CAH. Since there was no evidence of significant heterogeneity among the studies (I^2^ = 0%, *P*<0.05), a fixed-effect model was used for analysis. The result of meta-analysis showed that the incidence of CAH was 0.43‱ [95%CI, (0.39‱, 0.48‱)], or 1/23 024 [95%CI, (1/25 757,1/20 815)]. We also performed a subgroup analysis of regional incidences, among them, the incidence in Zhejiang, Guangdong, Hubei and Shaanxi province was lower than the national incidence; but in other regions, it was higher than the national incidence (see **Figures 2** and **3**).

#### Screening Positive Rate

**Figure 4:**
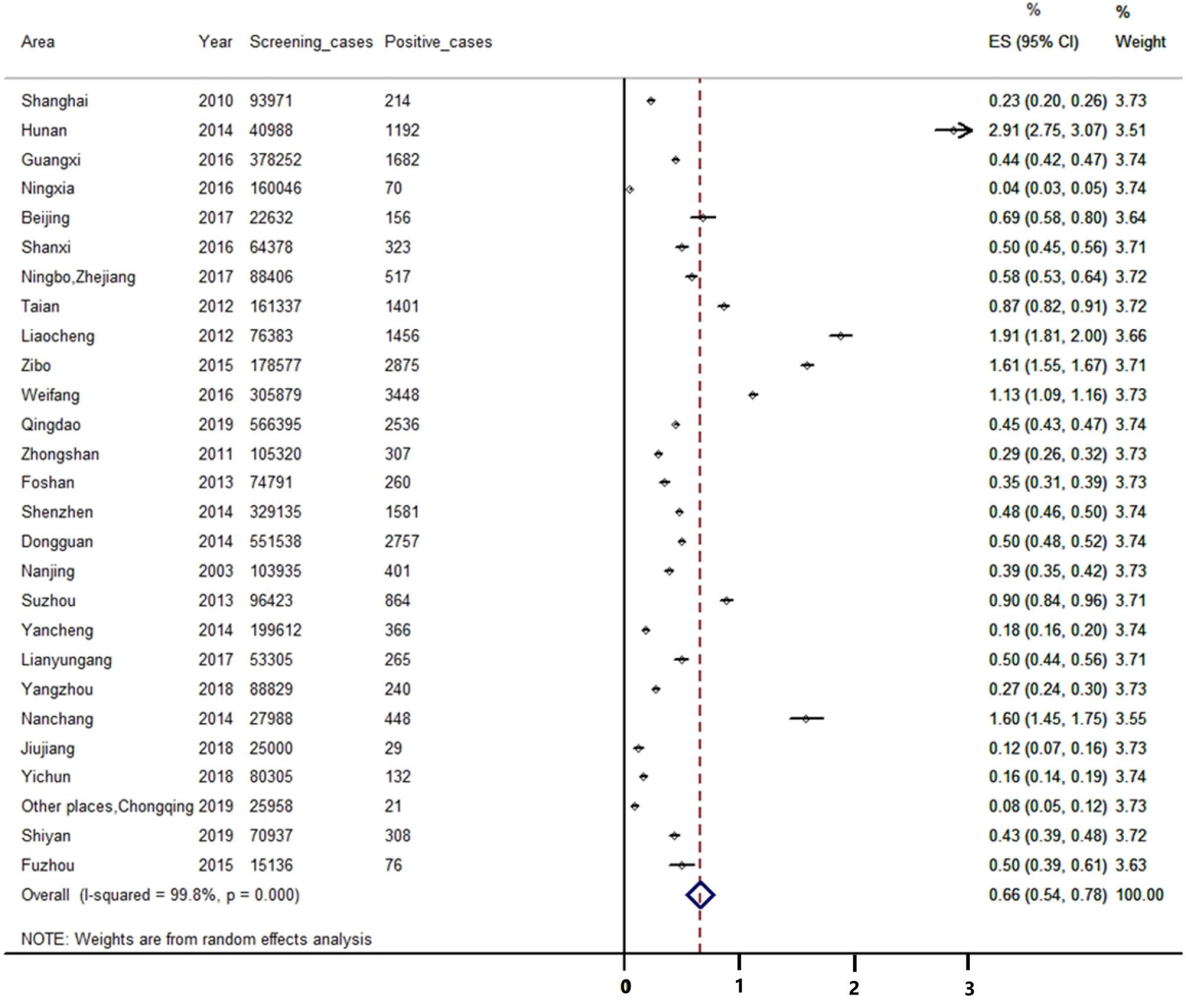
Meta-analysis of the positive rate of CAH screening.

In the included studies, 27 studies reported the positive rate of CAH screening. We found that 3 985 456 newborns were screened in these studies and 23 925 cases were considered as suspected positive cases. As I^2^> 50%, we used a random effect model for analysis. The result of the meta-analysis showed that the positive rate of CAH screening in China was 0.66% [95%CI, (0.54%, 0.78%)] (see **Figure 4**).

#### Recall Rate of Positive Cases

**Figure 5:**
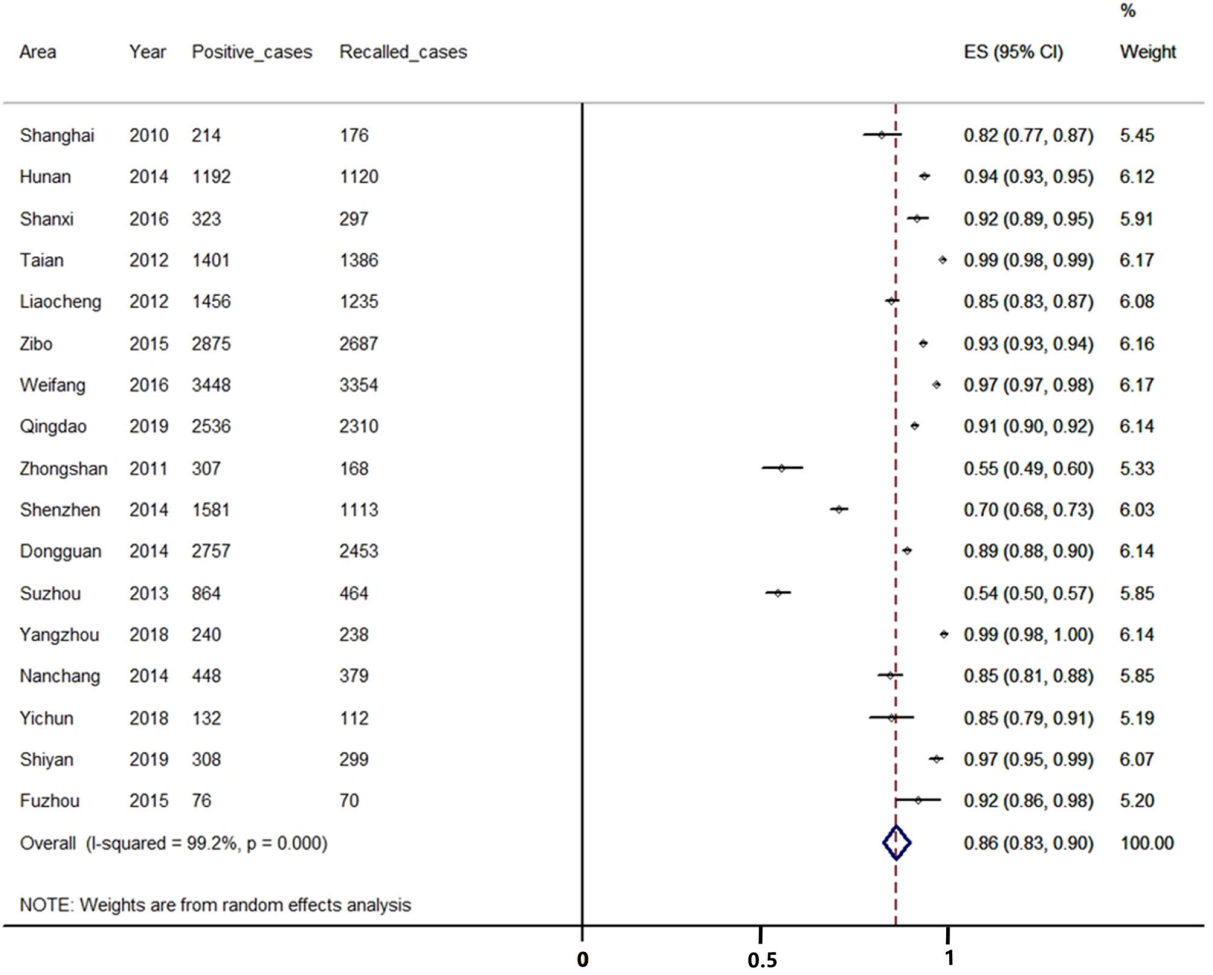
Meta-analysis of the recalled rate of positive cases during CAH screening.

In the included studies, 17 studies reported the recall rate of suspected positive cases. We found that 20 158 suspected positive cases were considered in our studies and 17 861 cases were successfully recalled, among which 135 cases were diagnosed with CAH (positive predictive value: 0.76%). As I^2^> 50%, we used a random effect model for analysis. The result of the meta-analysis showed that the recall rate of positive cases in China was 86.17% [95%CI, (82.70%, 89.64%)] (see **Figure 5**).

#### Publication Bias Across Studies

**Figure 6:**
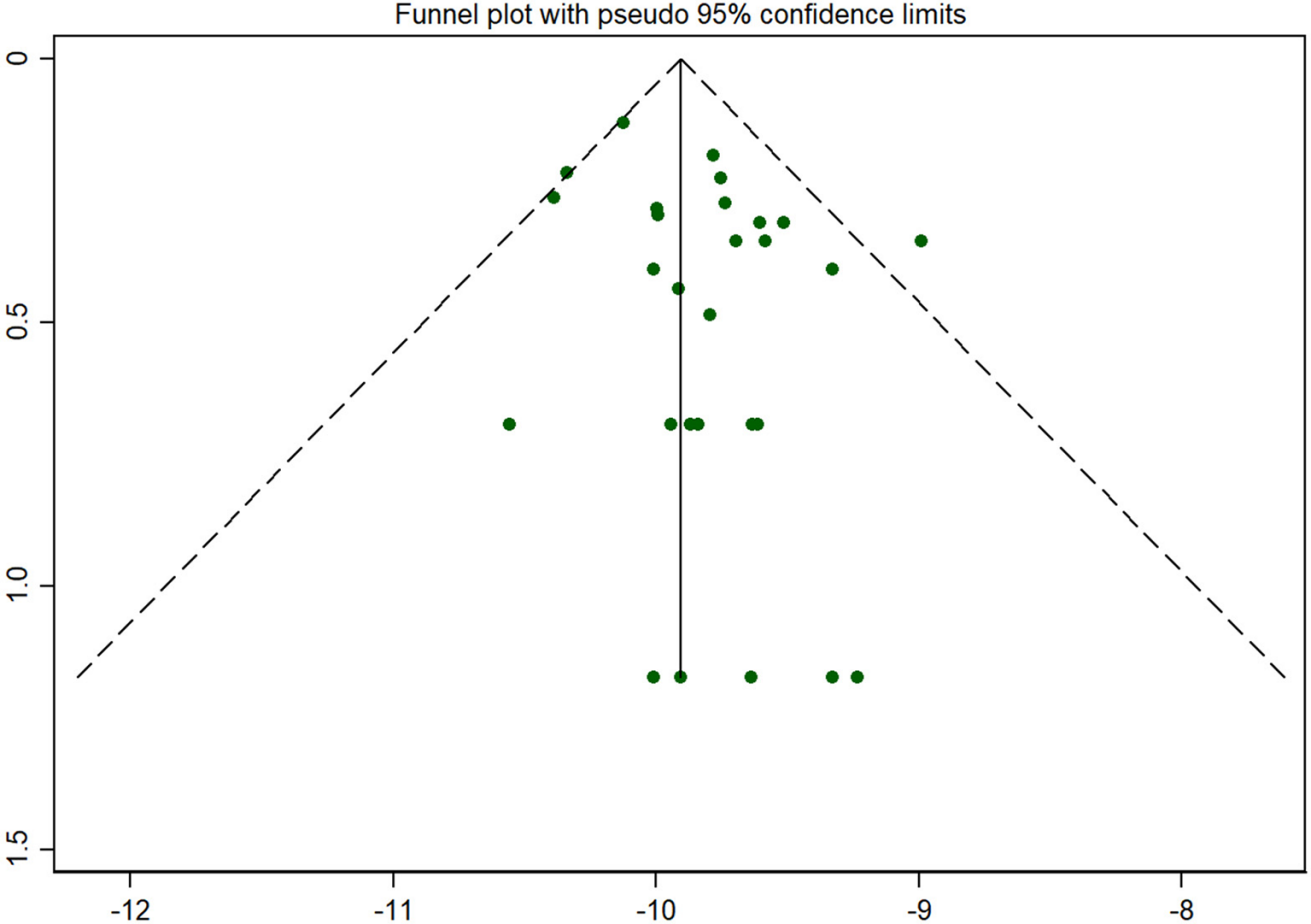
Funnel plot for publication bias. Note: As for the main indicator (the incidence of CAH), the funnel plot showed that all the included studies were symmetrically distributed in the triangle area, which meant that they were less affected by publication bias.

Publication bias was shown by a funnel plot and evaluated by the Begg’s test using Stata 12.0 software. As for the main indicator (the incidence of CAH), the funnel plot showed that all the included studies were symmetrically distributed in the triangle area (see **Figure 6**), which meant that they were less affected by publication bias. Begg’s test showed *P*=0.204 for the incidence of CAH, as for the other indicators, no publication bias was found between them (*P* value of the positive rate and the recall rate were 0.868 and 0.902, respectively).

## Discussion

Our meta-analysis included 41 studies on CAH screening of newborns in China, including approximately 7.85 million newborns, which is the most comprehensive and systematic analysis of CAH screening in the world. Due to the large sample size, representative population distribution, and no publication bias in the literature, the results of our study are objective and reliable.

According to the degree of enzyme deficiency, classical CAH represented by 21-OHD can be divided into two types: SW (about 75%) and SV (about 25%) type. In our study, we found that the ratio of SW to SV type is 3.25:1, which is consistent with previous literature reports. Due to the almost complete lack of enzyme activity, SW type will present with more typical clinical symptoms, just as in our study, 17-OHP levels of SW type were significantly higher than that of SV type. Theoretically, the incidence of autosomal recessive genetic disease such as CAH is the same for males and females. However, it’s interesting that the CAH screening results in our study showed that the incidence of CAH in males was much higher than that in females (1.92:1). Such a difference has not been reported elsewhere, so we need to be cautious about this result. Possible explanations based on China’s national conditions need to be considered: Firstly, there was a serious gender imbalance in China, and the screening data also showed the proportion of males was much higher than that of females (1.10:1); and Chinese parents will pay more attention to boys, such that the recall rate of positive boys may be higher than that of girls. Another explanation might be that some females were clinically diagnosed due to ambiguous genitalia after birth or even during pregnancy, making sample screening unnecessary (72 hours after birth). Also, since male patients with CAH tend to have higher levels of 17-OHP than the female, they may be more sensitive to CAH screening. A study of 220 000 newborns in the United States (45) showed that the sensitivity of newborn screening for male infants is 80%, while the female is only 60%. These reasons may account for the differences in our study results compared with previously reported studies, but we have to interpret these results objectively. Large-scale and prospective research will help verify our analysis.

According to relevant screening statistics, there are obvious racial and regional differences in the incidence of CAH. The global incidence of CAH is about 1/14 000-1/18 000 (2), among which, Japan is 1/19 859 (46), New Zealand is 1/26 727 (47), France is 1/15 699 (48), and Sweden is 1/14 260 (49). Our study has shown that the incidence of CAH in China is 0.43‱ (0.39‱, 0.48‱), that is 1/23 024 (1/25 757, 1/20 815), which is lower than most countries in Europe and America, and close to Asia-Pacific countries such as Japan and New Zealand. In China, the incidence in Zhejiang, Guangdong, Hubei and Shaanxi province is lower than the national incidence; but in other regions, it is higher than the national incidence. However, because of the inequality in medical provision, particularly the under-developed health-care in western China, missed diagnosis and misdiagnosis may occur, which may render the incidence of CAH screening lower than the actual incidence.

At present, the DELFIA or ELISA method is extensively used in China to detect the 17-OHP concentration in dried blood spots for CAH screening. These methods have strong specificity and high sensitivity, and provide a good technical accuracy for the screening work. In our study, 27 studies included 3 985 456 newborns reported the positive rate of CAH screening, among which 23 925 cases were considered as presumptive positive cases. Our study found that the positive rate (0.66%) of primary screening for CAH was much higher than the incidence rate (0.43‱), meaning that the current screening method may have a high false positive rate and a low positive predictive value. 17-OHP is a sensitive indicator for screening for CAH, but the setting of an appropriate cut-off value is difficult especially in premature and low birth weight infants which may give controversial screening results. Secondary screening such as liquid chromatography-tandem mass spectrometry (LC-MS/MS) can greatly improve the sensitivity and specificity of CAH screening. For example, within 3 years of using LC-MS/MS as secondary screening, the positive predictive value of the CAH screening in Minnesota, the United States, increased from 0.64% to 7.3%. However, LC-MS/MS can be used only as a supplement to primary screening and cannot completely replace the current methods.

In addition, compared with screening for congenital hypothyroidism and phenylketonuria, the screening coverage and recall rate of CAH are still very low. Our study included 17 studies, 20 158 suspected positive cases were considered, but only 17 861 cases were successfully recalled. Meta-analysis showed that the recall rate was only 86.17% (82.70%, 89.64%). This suggests that about 14% of newborns with positive results failed to be recalled, and there was a risk of delayed diagnosis or even missed diagnosis. The Southeast region accounts for the vast majority of China’s population, but the recall of newborns may be hampered by the complex population structure in southeast China, which has a large number of migrants and high mobility. We believe that because of the low awareness of some screening institutions and insufficient diagnostic level of some underdeveloped areas, CAH screening and diagnosis may be limited. Therefore, we should endeavor to raise public awareness of CAH to improve cooperation with the CAH screening program.

## Conclusion

Through the systematic analysis of the results of CAH screening for newborns in China, we have obtained a relatively accurate incidence of CAH in China (1/25 757, 1/20 815). In addition, we have established some interesting clinical characteristics of CAH, such as the ratio of different types and gender of CAH as well as their 17-OHP levels, which will provide valuable data for the screening and diagnosis of CAH in the future. However, we also realize that there are still some problems with CAH screening at present, such as the insufficient screening coverage in China, the difficulty of recalling positive cases, the imperfect setting of the 17-OHP cut-off value and the low positive predictive value, which will guide our future work in CAH neonatal screening. In summary, our study involving the largest number of babies on the incidence and regional characteristics of CAH provides data which suggest that improving laboratory testing capacity and equity of the CAH screening service throughout China should improve survival and quality of life for all.

## Data Availability Statement

The original contributions presented in the study are included in the article/**Supplementary Material**. Further inquiries can be directed to the corresponding author.

## Author Contributions

YL conceptualized and designed the study. ZL supervised the data collection, reviewed the analyses and wrote all versions of the manuscript. LH, CD, CZ, MZ and XL coordinated and supervised data collection, critically reviewed the manuscript and approved the final manuscript as submitted. All authors contributed to the article and approved the submitted version.

## Disclaimer

Frontiers Media SA remains neutral with regard to jurisdictional claims in published maps and institutional affiliations.

## Conflict of Interest

The authors declare that the research was conducted in the absence of any commercial or financial relationships that could be construed as a potential conflict of interest.

## Abbreviations

CAH, congenital adrenal hyperplasia; SW, salt wasting; SV, simple virilization; 17-OHP, 17-hydroxyprogesterone; 21-OHD, 21-hydroxylase deficiency; DELFIA, dissociation-enhanced lanthanide fluorescence immunoassay; ELISA, enzyme-linked immunosorbent assay; LC-MS/MS, liquid chromatography-tandem mass spectrometry; AHRQ, Agency for Healthcare Research and Quality of America; CI, confidence interval.
