# Supplementary material for: Analysis of the Screening Results for Congenital Adrenal Hyperplasia Involving 7.85 Million Newborns in China: A Systematic Review and Meta-Analysis
Source: Front Endocrinol (Lausanne). 2021 Apr 23;12:624507. doi: 10.3389/fendo.2021.624507 (PMC8104032; doi:10.3389/fendo.2021.624507)
Supplement: Supplementary file 2 [file Table_2.docx]

**Supplementary Table 2.** Quality assessments of included studies using an 11-item checklist recommended by AHRQ

| Area | | | ① | ② | ③ | ④ | ⑤ | ⑥ | ⑦ | ⑧ | ⑨ | ⑩ | ⑪ | AHRQ scores |
| --- | --- | --- | --- | --- | --- | --- | --- | --- | --- | --- | --- | --- | --- | --- |
| Province | | City |  |  |  |  |  |  |  |  |  |  |  |  |
| Taiwan(4) | | | 1 | 1 | 1 | 1 | 1 | 1 | 0 | 0 | 0 | 1 | 1 | 8 |
| Shanghai(5) | | | 1 | 1 | 1 | 1 | 1 | 1 | 0 | 1 | 0 | 1 | 1 | 9 |
| Hunan(6) | | | 1 | 1 | 1 | 1 | 1 | 1 | 0 | 1 | 0 | 1 | 1 | 9 |
| Guangxi(7) | | | 1 | 0 | 1 | 1 | 1 | 1 | 0 | 1 | 0 | 1 | 0 | 7 |
| Ningxia(8) | | | 1 | 1 | 1 | 1 | 1 | 1 | 0 | 1 | 0 | 1 | 1 | 9 |
| Beijing(9) | | | 1 | 0 | 1 | 1 | 0 | 1 | 0 | 1 | 0 | 1 | 1 | 7 |
| Sichuan(10) | | | 1 | 1 | 1 | 1 | 1 | 1 | 0 | 1 | 0 | 1 | 1 | 9 |
| Shanxi(11) | | | 1 | 1 | 1 | 1 | 1 | 1 | 0 | 1 | 0 | 1 | 1 | 9 |
| Zhejiang | Ningbo(12) | | 1 | 1 | 1 | 1 | 1 | 1 | 0 | 0 | 0 | 1 | 1 | 8 |
|  | Others(13) | | 1 | 0 | 1 | 1 | 0 | 1 | 0 | 1 | 0 | 1 | 0 | 6 |
| Shandong | Jinan(14) | | 1 | 1 | 1 | 1 | 1 | 1 | 0 | 0 | 0 | 1 | 1 | 8 |
|  | Taian(15) | | 1 | 1 | 1 | 1 | 1 | 1 | 0 | 0 | 0 | 1 | 1 | 8 |
|  | Liaocheng(16) | | 1 | 0 | 1 | 1 | 0 | 1 | 0 | 0 | 0 | 1 | 0 | 5 |
|  | Linyi(17) | | 1 | 1 | 1 | 1 | 1 | 1 | 0 | 0 | 0 | 1 | 0 | 7 |
|  | Heze(18) | | 1 | 0 | 1 | 1 | 0 | 0 | 0 | 0 | 0 | 1 | 1 | 5 |
|  | Zibo(19) | | 1 | 1 | 1 | 1 | 1 | 1 | 0 | 1 | 0 | 1 | 1 | 9 |
|  | Weifang(20) | | 1 | 1 | 1 | 1 | 1 | 1 | 0 | 0 | 0 | 1 | 1 | 8 |
|  | Rizhao(21) | | 1 | 0 | 1 | 1 | 0 | 1 | 0 | 0 | 0 | 1 | 0 | 5 |
|  | Qingdao(22) | | 1 | 1 | 1 | 1 | 1 | 1 | 0 | 1 | 0 | 1 | 1 | 9 |
| Guangdong | Zhongshan(23) | | 1 | 1 | 1 | 1 | 1 | 1 | 0 | 1 | 0 | 1 | 1 | 9 |
|  | Foshan(24) | | 1 | 1 | 1 | 1 | 1 | 1 | 0 | 1 | 0 | 1 | 1 | 9 |
|  | Shenzhen(25) | | 1 | 1 | 1 | 1 | 1 | 1 | 0 | 1 | 0 | 1 | 1 | 9 |
|  | Dongguan(26) | | 1 | 1 | 1 | 1 | 1 | 1 | 0 | 1 | 0 | 1 | 1 | 9 |
|  | Heyuan(27) | | 1 | 1 | 1 | 1 | 1 | 1 | 0 | 0 | 0 | 1 | 0 | 7 |
| Jiangsu | Nanjing(28) | | 1 | 1 | 1 | 1 | 1 | 1 | 0 | 0 | 0 | 1 | 1 | 8 |
|  | Wuxi(29) | | 1 | 1 | 1 | 1 | 1 | 1 | 0 | 0 | 0 | 1 | 1 | 8 |
|  | Changzhou(30) | | 1 | 1 | 1 | 1 | 1 | 1 | 0 | 0 | 0 | 1 | 1 | 8 |
|  | Suzhou(31) | | 1 | 1 | 1 | 1 | 1 | 1 | 0 | 0 | 0 | 1 | 1 | 8 |
|  | Yancheng(32) | | 1 | 1 | 1 | 1 | 1 | 1 | 0 | 1 | 0 | 1 | 1 | 9 |
|  | Lianyungang(33) | | 1 | 1 | 1 | 1 | 1 | 1 | 0 | 1 | 0 | 1 | 1 | 9 |
|  | Yangzhou(34) | | 1 | 1 | 1 | 1 | 1 | 1 | 0 | 1 | 0 | 1 | 1 | 9 |
| Jiangxi | Nanchang(35) | | 1 | 1 | 1 | 1 | 1 | 1 | 0 | 1 | 0 | 1 | 1 | 9 |
|  | Jiujiang(36) | | 1 | 1 | 1 | 1 | 1 | 1 | 0 | 0 | 0 | 1 | 0 | 7 |
|  | Yichun(37) | | 1 | 1 | 1 | 1 | 1 | 1 | 0 | 1 | 0 | 1 | 1 | 9 |
| Chongqing | Yuzhong(38) | | 1 | 0 | 1 | 1 | 0 | 1 | 0 | 0 | 0 | 1 | 0 | 5 |
|  | Others(39) | | 1 | 0 | 1 | 1 | 0 | 0 | 0 | 1 | 0 | 1 | 0 | 5 |
| Liaoning | Shenyang(40) | | 1 | 1 | 1 | 1 | 1 | 1 | 0 | 0 | 0 | 1 | 1 | 8 |
| Hubei | Shiyan(41) | | 1 | 0 | 1 | 1 | 1 | 1 | 0 | 0 | 0 | 1 | 0 | 6 |
| Shanxi | Baoji(42) | | 1 | 1 | 1 | 1 | 1 | 1 | 0 | 1 | 0 | 1 | 1 | 9 |
| Fujian | Fuzhou(43) | | 1 | 0 | 1 | 1 | 0 | 0 | 0 | 0 | 0 | 1 | 1 | 5 |
| Yunan | Kunming(44) | | 1 | 0 | 1 | 1 | 0 | 1 | 0 | 0 | 0 | 1 | 1 | 6 |

Note: NM, Not Mentioned.

An 11-item checklist recommended by Agency for Healthcare Research and Quality of America (AHRQ)：

①Define source of information (survey, record review);

②List inclusion and exclusion criteria for exposed and unexposed subjects (cases and

controls) or refer to previous publications;

③Indicate time period used for identifying patients;

④Indicate whether or not subjects were consecutive if not population-based;

⑤Indicate if evaluators of subjective components of study were masked to other aspects of the status of the participants;

⑥Describe any assessments undertaken for quality assurance purposes (e.g., test/retest of primary outcome measurements);

⑦Explain any patient exclusions from analysis;

⑧Describe how confounding was assessed and/or controlled;

⑨If applicable, explain how missing data were handled in the analysis;

⑩Summarize patient response rates and completeness of data collection;

⑪Clarify what follow-up, if any, was expected and the percentage of patients for which incomplete data or follow-up was obtained.
